# 

**Published:** 1819-08

**Authors:** 


### MONTHLY CATALOGUE OF MEDICAL BOOKS.

A Treatise on the Physiology and Diseases of the Ear. By John Harrison Curtis, Esq. Second Edition, with considerable additions and improvements. 8vo. 7s. 6d.

A complete Treatise on the Symptoms, Effects, and Nature of the Treatment of Syphilitic Disorders. Translated from the seventh French Edition of F. Swediaur, M.D. 2 vols. 8vo. 1l. 4s.

1

Observations on the Middle and Lower Classes in the North of Ireland; and on the late Epidemic Disorder, as it prevailed in an extensive District of that Country. By Francis Rogan, M.D. Physician to the Strabane Fever Hospital, &c. 8vo. 5s. 6d.

Discourses on the Elements of Therapeutics and Materia Medica. By N. Chapman, M.D. &c. 2 vols. 8vo.

History and Description of an Epidemic Fever, commonly called Spotted Fever, as it appeared at Gardiner, in the United States. By E. Hale, M.D. 8vo.

An Explanation of the Real Process of the Spontaneous Evolution of the Fœtus, &c. &c. By John C. Douglas, M.D. 8vo. 2s. 6d.

